# Combined Surgical and Nonsurgical Endodontic Therapy in the Treatment of Dens Invaginatus Type 3: A Case Report

**DOI:** 10.5005/jp-journals-10005-1018

**Published:** 2009-12-26

**Authors:** Nikhil Marwah, Puneet Goenka, Anant Gopal Nigam, Prathima GS

**Affiliations:** 1 Reader, Department of Pedodontics and Preventive Dentistry, Mahatma Gandhi Dental College, Jaipur, Rajasthan, India; 2 Senior Lecturer, Department of Pedodontics and Preventive Dentistry, Mahatma Gandhi Dental College, Jaipur, Rajasthan India; 3 Reader, Department of Pedodontics and Preventive Dentistry, Mahatma Gandhi Dental College, Jaipur, Rajasthan, India; 4 Professor and Head, Department of Pedodontics and Preventive Dentistry, Mahatma Gandhi Dental College, Jaipur, Rajasthan India

**Keywords:** Dens invaginatus, endodontic therapy, surgery, incisors.

## Abstract

An accurate understanding of the morphology of the root canal system is a prerequisite for successful root canal treatment. Invaginated teeth have a complex root canal configuration that cannot be instrumented effectively. Correct diagnosis and treatment planning are fundamental to treatment of dens invaginatus. Periapical surgery is indicated in cases where a nonsurgical approach fails. A case of dens invaginatus type 3 in a maxillary lateral incisor with a periapical lesion and its successful treatment by these combined methods is reported.

Dens invaginatus (DI), also known as "dens in dente", "dilated composite odontome" or "gestant odontoma", is a developmental anomaly resulting from invagination of a portion of the crown (enamel organ) during morphodifferentiation. 1-4 Salter^5^ first described anatomical anomaly in 1855 as "a tooth within a tooth." The etiology is controversial and remains unclear^4^ however, most authors concur the above mentioned reason. Other theories include an incomplete lateral fusion of two germs, the distortion of the enamel organ during tooth development, abnormal pressure from the surrounding tissues during tooth formation, the constriction of the dental arch in the enamel organ and a retardation or acceleration of growth of the internal enamel epithelium.^6^-^8^

Dens invaginatus with a frequency of 0.04-10% is a rare dental malformation.^9^ The most affected permanent teeth are the maxillary lateral incisors, frequently bilateral (43 percent),^10^ followed by central incisors, canines, premolars and molars.^6^ It also can appear in primary teeth.^11^

Of the three classifications designed for dens invaginatus, clinicians most commonly use the one proposed by Oehlers.^8^

- *Type I:* An enamel-lined invagination within the crown and not extending beyond the cementoenamel junction (CEJ);
- *Type II:* The enamel invagination into the root, beyond the CEJ, ending as a blind sac;
- *Type III:* The extension of the enamel-lined invagination through the root to form an additional apical or lateral foramen; usually, there is no direct communication with the pulp.

The purpose of this article is to describe the use of combined endodontic therapy and surgery in the treatment of Type 3 dens invaginatus in a maxillary lateral incisor.

## CASE REPORT

**Fig. 1:**
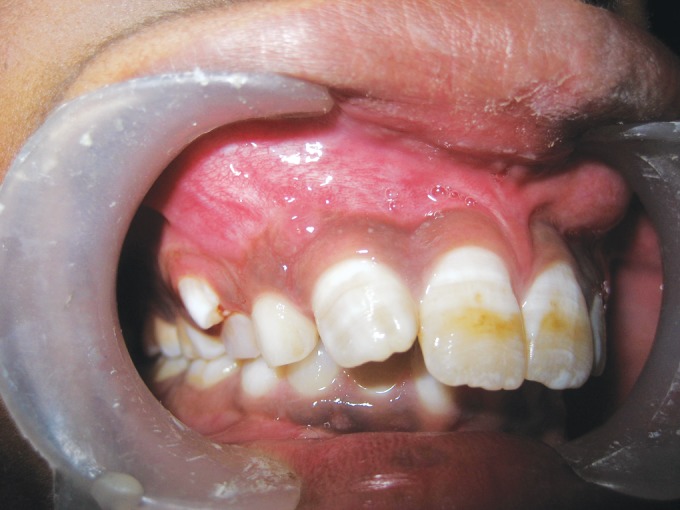
Clinical presentation

**Fig. 2:**
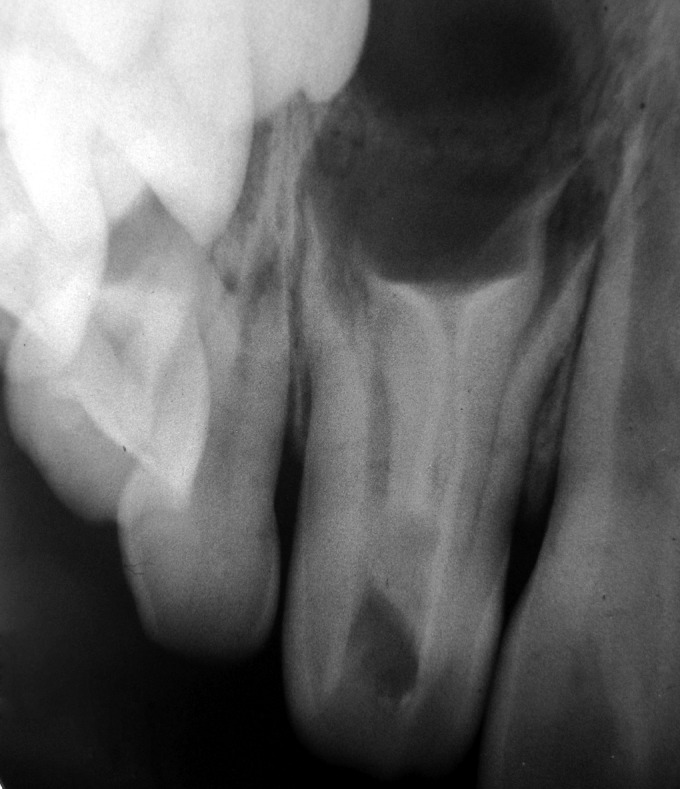
IOPA showing Dens invaginatus i.r.t. upper right lateral incisor

**Fig. 3:**
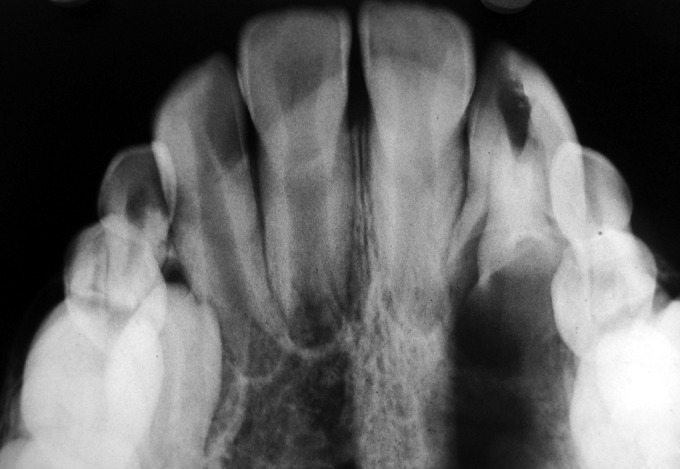
Occlusal radiograph

A ten years old boy reported to pediatric dental clinic with the chief complain of repeated swelling and pain in the anterior region of the upper jaw since 3 months. The pain was of severe type and nonradiating in nature. Pain subsided on taking analgesics. The swelling subsided in 3-4 days on its own. Patient gave a negative history for any kind of trauma. Patient did not complain of any discharge in the oral cavity. Patient’s medical and family history was uncontributory. On examination no carious teeth were found in the oral cavity. A bulge was seen in the vestibule over the upper right deciduous canine which was assumed to be due to erupting permanent canine (Fig. 1). The tooth under suspicion was the upper right lateral incisor. A radiograph was advised for this tooth. The IOPA revealed a dens invaginatus with an extended area of periapical radiolucency (Figs 2 and 3). On further examination the tooth was nontender to percussion and firm in oral cavity. Periodontal probing affirmed normal attachment, with no reading greater than three millimeters. The tooth responded negatively to vitality testing, though the adjacent central incisor responded within normal limits. The color of the tooth did not differ from that of the adjacent teeth, and the total number of teeth in the anterior region was normal. An invagination with two other canals extending up to the apical region was evident on the radiograph. The anatomy of the tooth was consistent with a type III dens invaginatus. The root apices appeared to be incomplete. A conservative approach was taken to treat the condition. The area was anesthetized and access cavity was prepared after rubber dam application. Three distinct openings were found in the floor of the pulp chamber. Working length was determined with the help of apex locator (Root ZX J. Morita, Kyoto, Japan) and radiographic method. The canals were shaped in a crowndown method using Gates Glidden drills and K-files (Maillefer, Ballaigues, Switzerland) and were irrigated copiously with 2.5% Na hypochlorite. The canals were filled with calcium hydroxide in a polyethylene glycol vehicle and the access cavity was restored with a temporary restorative material.

**Fig. 4:**
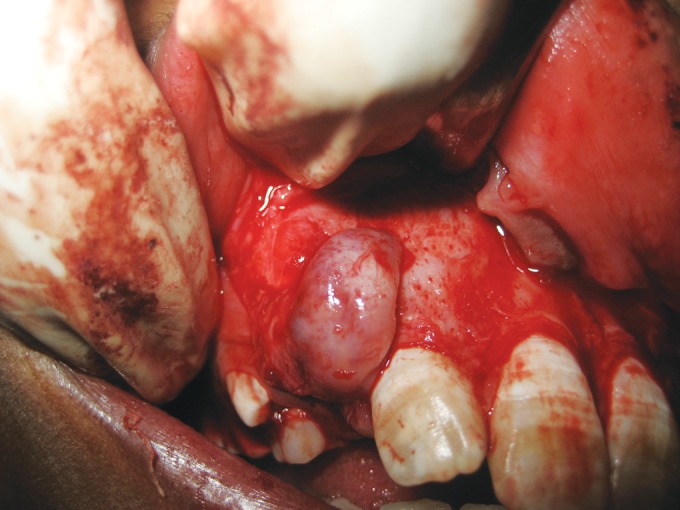
Intraoperative view

**Fig. 5:**
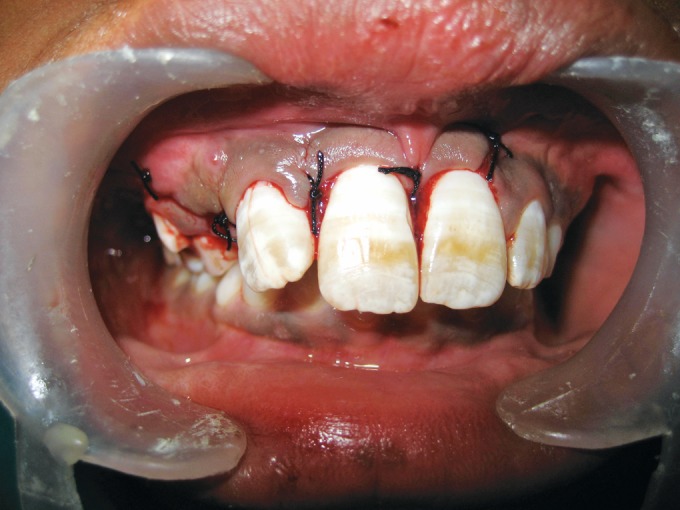
Postoperative suturing

**Fig. 6:**
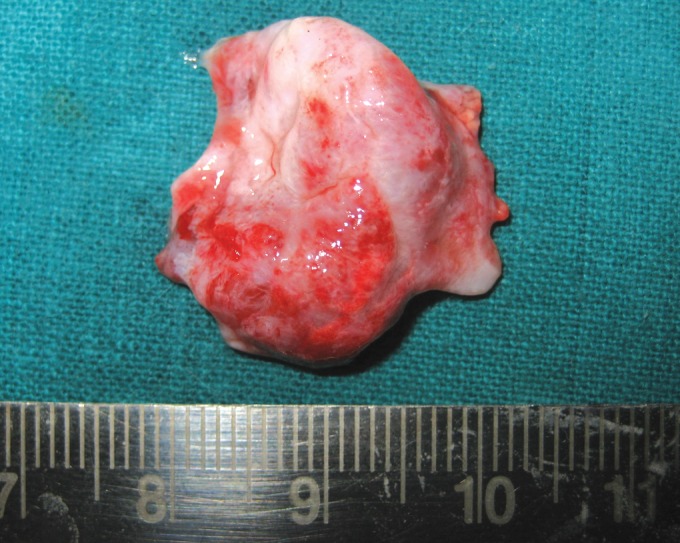
Enucleated cyst lining

**Fig. 7:**
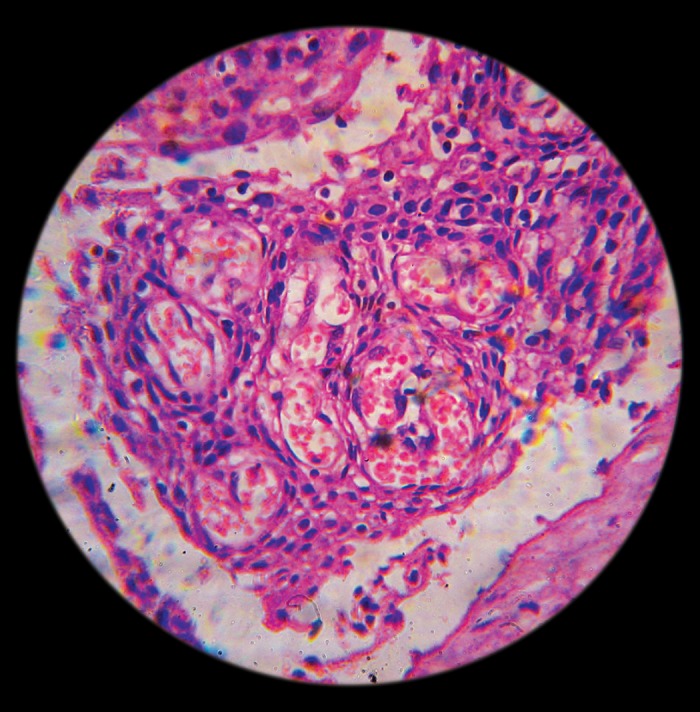
Photomicrograph showing the Rushton bodies

**Fig. 8:**
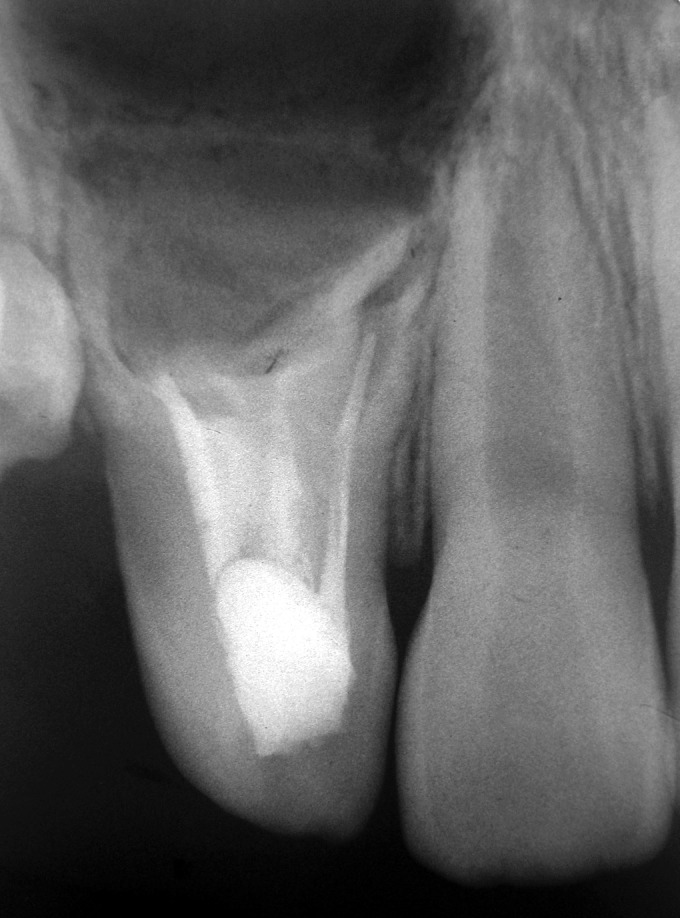
Follow-up radiograph after 1 year

On subsequent follow-up it was found that the symptoms of the patient did not resolve. Thus it was decided to perform the surgical treatment for the case. After the preliminary blood investigations the surgical procedure was planned. The root canals were irrigated properly with 5.25% sodium hypochlorite and finally obturated with gutta-percha and zinc oxide eugenol sealer with the lateral condensation technique. The area was anesthetized and a full thickness mucoperiosteal flap was raised on the buccal side. A bony defect was evident in the apical region of the lateral incisor. The defect was enlarged slightly to gain access to the apical portion(Fig. 4). The defect lining was carefully enucleated. A lining of dimension 2.5 × 2.3 cm was excised (Fig. 5). The bony defect was curetted properly and irrigated with normal saline. After complete debridement and achievement of hemostasis, retrograde filling was done in the root canal apices with self cure type II Glass Ionomer Cement. Interrupted sutures were placed with nonresorbable suture material (silk) (Fig. 6). The patient was advised antibiotics Amoxicillin 250 mg tid for five days and analgesics Ibuprofen 200 mg tid for 3 days. The patient was recalled after 7 days for suture removal. Further the patient was kept on monthly follow-up. The cyst lining was sent for histopathological examination which revealed a stratified squamous cell lining with no atypical cells present. The subepithelial tissue was infiltrated by subacute inflammatory infiltrate suggestive of a radicular cyst (Fig. 7). The patient has been found to be symptomless with resolving periapical radiolucency for last one year of follow-up (Fig. 8).

## DISCUSSION

Dens-in-dente is a rare anomaly of tooth which has its clinical implication due to the complications caused in response to the anatomical alteration of the tooth. Teeth with dens invaginatus are prone to early development of caries and subsequent necrosis of the pulp. As a matter of prevention, clinicians are encouraged to seal the invagination prophylactically with resin.^12^ In cases in which the bacterial invasion has reached the pulp and necrosis is established, nonsurgical root canal treatment remains the treatment of choice. Depending on the type of malformation and the communication of the invaginationwith the pulp, the clinician may confine the endodontic therapy to the invaginated portion and, as a result, preserve pulp vitality.^13^,^14^ However, in most cases, the endodontic treatment must include both the invagination and the root canals.^15^,^16^ The task can become difficult, considering the anatomical variations that a dens invaginatus may present within the root canal system.

The treatment of invaginated teeth is a complex procedure. They present a complex root canal configuration that is often not possible to instrument completely. As a result, these teeth should be selected for combined orthograde and surgical treatment.^12^,^17^ Endodontic surgery is indicated for cases of severe forms of dens invaginatus with a prominent periapical involvement.^18^ In other cases, combined treatment may be necessary, that is, endodontic therapy followed by endodontic surgery, because of the complexity of the root morphology. The surgery will provide an additional retrograde seal to the root canals, which may remain a source of irritation.^19^ Thus it can be said that it is the complexity of the root canal system not the size of the periradicular lesion that dictate the treatment procedure or influence the treatment outcomes of nonsurgical root canal therapy. Thus, if nonsurgical endodontic therapy fails, a combined approach with apical surgery may be indicated. In the present case also initially effort was made to treat the case conservatively through a nonsurgical approach. It was only when the nonsurgical method failed the surgical procedure was performed.

Since thorough instrumentation of root canal is not possible due to the complex root canal morphology, an intracanal medicament may be required to disinfect the canal system. Calcium hydroxide is considered as an ideal material for intracanal dressing. Its high alkalinity is considered responsible for the disinfection caused by it.

Clinically, unusual crown morphology or a deep lingual groove may lead us to suspect a tooth with DI, but affected teeth may also show no clinical signs of any malformation, as in the present case. In that situation radiographic examination plays the key role. If one tooth is affected, a contralateral tooth must also be checked. Characteristically, the DI presents as a deep invagination in the lingual or occlusal pit area. The invagination area is separated from the pulpal tissues with a thin layer of dentin and frequently communicates with the oral cavity, allowing the entry of irritants and microorganisms, which usually leads to dental caries or infection and necrosis of the pulpal tissue and then to periodontal or periapical abscess with continuous ingress. Histological examination shows that while the enamel covering the invagination is defective, the enamel and dentin tissues of the outer tooth are normal and not defective. The hypomineralized nature of the enamel covering of the invagination, incomplete enamel lining and existence of channels between the invagination and the pulp are also within the possible causes of the bacterial invasion.^20^

There have been reports on the concomitant presence of DI with other dental anomalies such as dentigonesis imperfecta, gemination, taurodontism, microdontia, supernumerary teeth and short roots and with some medicaldental syndromes.^1^,^4^ Thus whenever dens invaginatus is encountered any other possible dental anomaly should be ruled out.

To conclude Dens invaginatus requires an early diagnosis and treatment as they are more prone to pulp pathosis resulting from bacterial ingress. The treatment ranges from prophylactic restoration of deep grove to extensive periradicular surgery in combination with endodontic therapy depending upon extent of involvement.
